# Reduced length of stay and hospitalization costs among inpatient hysterectomy patients with postoperative pain management including IV versus oral acetaminophen

**DOI:** 10.1371/journal.pone.0203746

**Published:** 2018-09-13

**Authors:** Ryan N. Hansen, An T. Pham, Elaine A. Boing, Belinda Lovelace, George J. Wan, Richard D. Urman

**Affiliations:** 1 University of Washington, School of Pharmacy, Seattle, Washington, United States of America; 2 Mallinckrodt Pharmaceuticals, Health Economics and Outcomes Research Department, Hampton, New Jersey, United States of America; 3 University of California San Francisco, School of Pharmacy, San Francisco, California, United States of America; 4 Harvard Medical School and Brigham and Women’s Hospital, Boston, Massachusetts, United States of America; Cleveland Clinic Lerner College of Medicine of Case Western Reserve University, UNITED STATES

## Abstract

**Objective:**

To compare the outcomes of hysterectomy patients who received standard pain management including IV acetaminophen (IV APAP) versus oral APAP.

**Methods:**

We performed a retrospective analysis of the Premier Database (January 2012 to September 2015) comparing hysterectomy patients who received postoperative pain management including IV APAP to those who received oral APAP starting on the day of surgery and continuing up to the third post-operative day, with no exclusions based on additional pain management. We compared the groups on length of stay (LOS), hospitalization costs, and average daily morphine equivalent dose (MED). The quarterly rate of IV APAP use for all hospitalizations by hospital was used as an instrumental variable in two-stage least squares regressions also adjusting for patient demographics, clinical risk factors, and hospital characteristics.

**Results:**

We identified 22,828 hysterectomy patients including 14,811 (65%) who had received IV APAP. Study subjects averaged 50 and 52 years of age, respectively in the IV APAP and oral APAP cohorts and were predominantly non-Hispanic Caucasians (≥60% in both cohorts). Instrumental variable models found IV APAP associated with 0.8 days shorter hospitalization (95% CI: -0.92 to -0.68, p<0.0001) and $2,449 lower hospitalization costs (95% CI: -$2,902 to -$1,996, p<0.0001). Average daily MED trended lower without statistical significance (-1.41 mg, 95% CI: -3.43 mg to 0.61 mg, p = 0.17).

**Conclusions:**

Compared to oral APAP, managing post-hysterectomy pain with IV APAP is associated with shorter LOS and lower total hospitalization costs.

## Introduction

Hysterectomy is the second most frequently performed major procedure among reproductive-aged women in the United States (U.S.), with approximately 600,000 surgeries annually [1]. Effective post-hysterectomy pain management is indispensable to shorten hospital length of stay (LOS), reduce complications, improve patient comfort and satisfaction, and reduce hospitalization costs [2]. Reaching these objectives is also essential to meet the Triple Aim set by the Institute for Healthcare Improvement (IHI) [3].

Post-hysterectomy pain management often includes patient-controlled analgesia and an opioid regimen [2]. However, opioid administration has been associated with a variety of adverse events [4] and increased health care utilization and costs [5]. Studies have demonstrated the benefits of multimodal analgesia to improve pain control and reduce the incidence of opioid-related adverse events [6, 7]. In addition, medical provider groups and accreditation bodies have supported a multimodal approach to improving outcomes across multiple surgical populations [8]. For instance, the American Society of Anesthesiologists (ASA) Task Force on Acute Pain Management recommends the use of multimodal pain management therapy whenever possible. Furthermore, ASA guidelines support that acetaminophen (APAP) should be considered as part of a postoperative multimodal pain management regimen [9].

In patients undergoing open abdominal hysterectomy, multimodal pain control has been associated with significantly reduced hospital stay compared to morphine alone [10].

Acetaminophen, available in oral, rectal, and intravenous (IV) formulations, is a common component of multimodal pain management that may include pharmaceutical and non-pharmaceutical treatments. Intravenous administration of APAP has some potential pharmacokinetic advantages (time to maximum concentration and overall maximum concentration) over oral and rectal administration. A pharmacokinetic study has demonstrated that the IV route results in an average 76% higher and maximum 256% higher mean plasma concentration than oral or rectal administration, as well as faster time to reach maximum plasma concentration (consistent with absorption delays for oral or rectal formulations). That study also demonstrated 75% higher area under the curve (AUC) in cerebrospinal fluid concentration of APAP over six hours compared to oral administration, while the IV APAP AUC was 142% higher than the rectal formulation [11]. In addition, concomitant use of APAP tablets and opioids may result in inadequate pain control and lead to potential health safety risks due to gastric accumulation of APAP from opioid induced gastric dysfunction [12]. There is also developing evidence regarding the potential interaction of morphine with oral APAP in the gastric compartment [13].

Numerous studies have identified factors that affect gastric motility and decrease absorption of oral medications in the perioperative period [14–19]. Specifically, a recent study found that APAP peak concentration and area under the plasma concentration-time curve were reduced when oral APAP was co-administered with IV morphine. However, when IV APAP was co-administered with IV morphine, pharmacokinetics of APAP were not impacted. Additionally, there was an abrupt increase in peak concentration and area under the curve following discontinuation of IV morphine in the oral APAP group [20]. When acetaminophen is co-administered with morphine, the IV formulation produces more predictable blood levels compared to the oral formulation, which shows increased inter-individual pharmacokinetic variability [20].

Differences in outcomes associated with the use of IV APAP have been studied against placebo as well as other active comparators, such as non-steroidal anti-inflammatory drugs (NSAIDs). A meta-analysis of IV APAP versus placebo studies reported that 10 of 14 studies found that IV APAP was associated with less opioid consumption, a lower proportion of patients rescuing, or increased time to first rescue [21]. In hysterectomy, a randomized clinical trial demonstrated that pain scores, postoperative morphine consumption, and LOS were reduced in patients receiving IV APAP compared to placebo [22]. When compared to NSAIDs, a study reported that IV APAP was safe and effective for mild to moderate postoperative pain and may be especially advantageous when surgical bleeding is an issue [23].

However, as noted in some literature reviews, limited research has explored the comparative effectiveness of IV versus oral APAP [24, 25]. A study conducted outside the U.S. found that IV APAP was associated with reduced use of opioids after cardiovascular surgery [26], but it is not clear how these results generalize to the U.S. population or other surgical groups. A recent small systematic review of trials directly comparing IV versus oral APAP was inconclusive [27]. This gap in knowledge prevents a comprehensive assessment of the clinical and economic evidence regarding the route of administration of APAP in the post-surgical population. In this study, we sought to compare outcomes of hysterectomy patients who received usual care pain management including IV APAP versus oral APAP in hospitals across the U.S.

## Materials and methods

We analyzed data from the Premier Database between January 1, 2012 and September 30, 2015 in order to perform a retrospective cohort study of hysterectomy patients [7]. Nearly 20% of annual U.S. inpatient discharges are contained in the Premier Database, with data from over 750 hospitals/healthcare systems that are both regionally and demographically diverse. The database is comprised of summary and detailed billing/service records, including costs and charges, as well as procedure (International Classification of Diseases, 9th Revision, Clinical Modification [ICD-9-CM] and Current Procedural Terminology) and diagnosis (ICD-9-CM) codes.

Patients who underwent an inpatient hysterectomy at a Premier hospital (ICD-9-CM Procedure Code: 68.31, 68.39, 68.41, 68.51, 68.59, 68.61, and 68.69) and received either IV or oral APAP beginning on the day of surgery and continuing for no more than two additional days were included in our study population. Billing service records for IV and oral APAP were used to classify exposure. Patients were separated into mutually exclusive groups based on their receipt of either IV or oral APAP. Recipients of both IV and oral APAP on any of the first three post-operative days were excluded from our analyses. Additionally, we excluded outpatient surgical procedures, but allowed other pain treatments in both groups. Our primary analysis compared the IV and oral APAP recipients among all hysterectomy patients. We also stratified our patients into three groups by surgical procedure type as laparoscopic hysterectomy (ICD-9-CM: 68.31, 68.41, 68.51, and 68.61), total abdominal hysterectomy (ICD-9-CM: 68.39 and 68.69), and total vaginal hysterectomy (ICD-9-CM: 68.59).

### Outcomes

We defined six outcomes of interest *a priori*: 1) hospitalization LOS, 2) hospitalization cost, 3) average daily opioid dose billed, and 4–6) complications that may be attributed to opioids: 4) nausea/vomiting, 5) respiratory depression (composite and stratified by diagnosis, administration of naloxone, and mechanical ventilation), and 6) constipation/bowel obstruction/ileus. The Premier database records the LOS and total hospitalization costs. We did not evaluate the individual costs of APAP by route of administration, and at an institutional level costs may vary based on contracting arrangements. Daily doses of opioids were calculated as morphine equivalent dose (MED) using the Centers for Disease Control and Prevention algorithm over the entire course of the admission and then averaged at the patient level [28]. We used ICD-9-CM diagnosis codes along with naloxone and mechanical ventilation service codes augmenting the respiratory depression diagnosis to identify complications attributed to each patient.

### Statistical analyses

Our first analyses were a descriptive comparison of the IV APAP and oral APAP hysterectomy patients on both patient (age, gender, race, All Patient Refined Diagnosis Related Groups Severity of Illness [APR-DRG SOI], APR-DRG Risk of Mortality [ROM]) and hospital characteristics (U.S. Region, bed count, urban/rural indicator, and academic medical center indicator). The APR-DRG SOI and ROM indices represent proprietary algorithms developed by 3M Corporation for risk adjustment of hospitalizations [29]. The Student’s t-test was used for continuous variables and the Chi-square test was used for categorical variables in order to determine statistically significant differences between IV APAP and oral APAP patients. We then computed the differences in each outcome, using the Student’s t-test for LOS, cost, and daily MED, and unadjusted logistic regression for the complications. We recognized that our pragmatic approach to allow for all other pain medications to be used could potentially influence our assessment of differences in outcomes and thus we also estimated the differences in other analgesics used by both type and route of administration. We also evaluated the cost outcome separated into Premier hospital department level categories.

We recognize that selection bias may influence traditional methods for controlling the estimation of differences in outcomes between patient groups who received IV and oral treatment with the same medication. Thus we performed an instrumental variable regression, using each hospital’s rate of IV APAP use for all admissions on a quarterly basis as an exogenous factor (instrument) in a two-stage linear regression. Instrumental variable models were estimated for LOS, total hospitalization cost, and opioid dose. The main independent variable in these models was the indicator for the use of IV APAP, and we instrumented that variable with the hospital’s use of IV APAP for all hospitalizations. Both stages of the regression included variables for all patient and hospital characteristics. Such techniques are not available for binary outcomes, and thus we performed multivariable regression, using all available patient and hospital covariates, for the complications without the instrumental variable adjustment.

We performed all analyses using the pooled hysterectomy patients, and then also surgical subgroup analyses (laparoscopic, open total hysterectomy, and vaginal hysterectomy) of the instrumental variable regressions. This study utilized HIPAA compliant de-identified data and was approved by the Human Subjects Division at the University of Washington by self-determination.” We conducted our statistical analyses using SAS for Windows, Version 9.3 (SAS Institute Inc., Cary, NC) and STATA 13 (StataCorp LP, College Station, TX).

## Results

**Fig 1:**
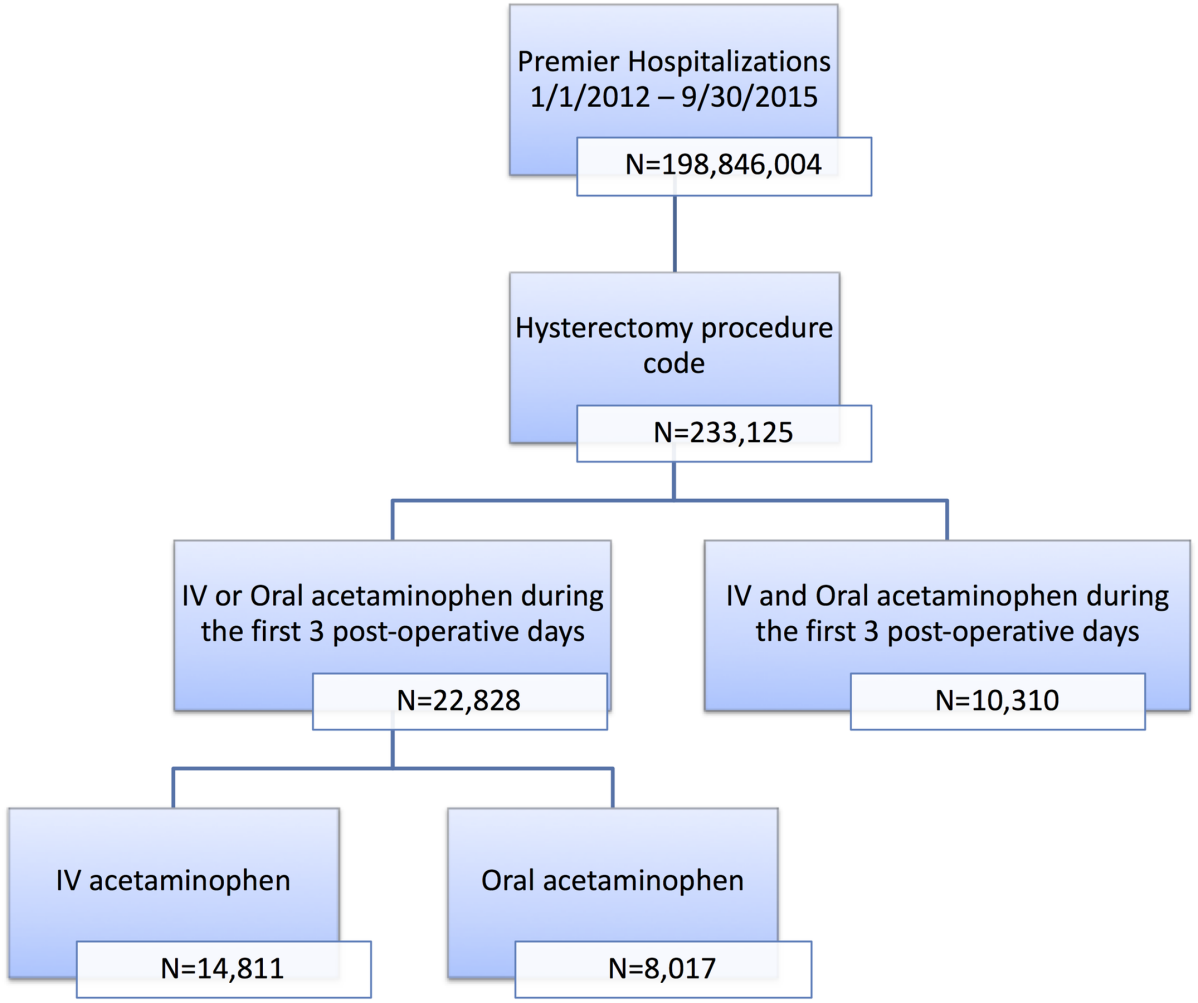
Sample selection.

A total of 22,828 hysterectomy patients were eligible for our study. Among those patients, 14,811 received IV APAP and 8,017 received oral APAP (Fig 1). There were 10,310 patients who received both IV and oral APAP that were excluded from our eligible population. Our study subjects were predominantly white (66.1% and 60.0% in the two groups) with minor APR-DRG SOI and ROM indices who were treated in large, urban hospitals (90.6% and 89.0% respectively). Due to the large sample size, there were statistically significant differences in all demographic and hospital characteristics (Table 1). When we stratified the population by type of hysterectomy these differences remained relatively consistent.

**Table 1:** Hysterectomy surgery population demographics.

	IV Acetaminophen* (n = 14,811)	Oral Acetaminophen* (n = 8,017)	p-value
Age, mean (S.D.)	49.7 (12.5)	52.3 (13.4)	<0.0001
Female, n (%)	14,810 (99.9)	8,016 (99.9)	0.7
Race, n (%)			<0.0001
White	9,792 (66.1)	4,809 (60.0)	
Black	2,310 (15.6)	899 (11.2)	
Other	2,695 (18.2)	2,297 (28.7)	
Unknown	14 (0.1)	12 (0.2)	
Surgery Type, n (%)			<0.0001
Laparoscopic	8,925 (60.3)	3,934 (49.1)	
Total Abdominal	2,678 (18.1)	2,268 (28.3)	
Vaginal	3,208 (21.7)	1,815 (22.6)	
APR-DRG Severity of Illness, n (%)			<0.0001
None	0 (0.0)	1 (0.0)	
Minor	9,945 (67.2)	4,539 (56.6)	
Moderate	4,236 (28.6)	2,657 (33.1)	
Severe	556 (3.8)	662 (8.3)	
Extreme	74 (0.5)	158 (2.0)	
APR-DRG Risk of Mortality, n (%)			<0.0001
None	0 (0.0)	1 (0.0)	
Minor	13,647 (92.1)	6,811 (85.0)	
Moderate	889 (6.0)	758 (9.5)	
Severe	216 (1.5)	334 (4.2)	
Extreme	59 (0.4)	113 (1.4)	
Emergent Admission, n (%)	2,270 (15.3)	952 (11.9)	<0.0001
Urban Hospital, n (%)	13,421 (90.6)	7,135 (89.0)	0.0001
Teaching Hospital, n (%)	6,326 (42.7)	4,629 (57.7)	<0.0001
Hospital Bed Count, mean (S.D.)	422.3 (232.1)	480.5 (262.4)	<0.0001
Year of Hospitalization, n (%)			<0.0001
2012	3,711 (25.1)	2,878 (35.9)	
2013	5,178 (34.9)	2,172 (27.1)	
2014	3,907 (26.4)	1,611 (20.1)	
2015	2,015 (13.6)	1,355 (16.9)	
Hospital Region, n (%)			<0.0001
Midwest	2,176 (14.7)	1,346 (16.8)	
Northeast	2,469 (16.7)	3,173 (39.6)	
South	8,111 (54.8)	2,036 (25.4)	
West	2,055 (13.9)	1,462 (18.2)	

*Subjects in each cohort were included regardless of additional pain management

### Inpatient length of stay

The mean unadjusted LOS was 0.8 days lower for IV APAP patients compared to oral APAP patients: 1.9 days (SD 2.2) vs. 2.7 days (SD 3.7), respectively, (95% CI: -0.9 to -0.7, p<0.0001) (Table 2). This difference was consistently estimated in the two-stage instrumental variable regression, with IV APAP associated with 0.8 days shorter hospitalization (95% CI: -0.9 to -0.7, p<0.0001) (Table 3).

**Table 2:** Unadjusted outcomes comparing IV and oral acetaminophen.

	IV Acetaminophen* (n = 14,811)	Oral Acetaminophen* (n = 8,017)	Difference (95% C.I.)	p-value
Length of Stay (days), mean (S.D.)	1.9 (2.2)	2.7 (3.7)	-0.8 (-0.9 to -0.7)	<0.0001
Hospitalization Cost ($), mean (S.D.)	9,867.3 (7,367.0)	12,215.5 (13,407.2)	-2,348.2 (-2,664.8 to -2,031.6)	<0.0001
Morphine Equivalent Dose (mg), mean (S.D.)	24.9 (44.7)	26.9 (31.7)	-1.7 (-2.7 to -0.7)	0.0011
*Complications*			Odds Ratio (95% C.I.)	
Bowel obstruction, n (%)	291 (2.0)	344 (4.3)	0.45 (0.38 to 0.52)	<0.0001
Nausea/vomiting, n (%)	260 (1.8)	225 (2.8)	0.62 (0.52 to 0.74)	<0.0001
Respiratory depression, n (%)	383 (2.6)	428 (5.3)	0.47 (0.41 to 0.54)	<0.0001

*Subjects in each cohort were included regardless of additional pain management

**Table 3:** Instrumental variable regressions comparing IV and oral acetaminophen patients*.

	Difference	95% Confidence Interval		p-value
Length of Stay (days)	-0.80	-0.92	-0.68	<0.0001
Hospitalization Cost ($)	-2,449.0	-2902.4	-1995.6	<0.0001
Morphine Equivalent Dose (mg)	-1.41	-3.43	0.61	0.17

*Two-stage least squares with quarterly rate of IV acetaminophen use at the hospital as the instrument. Adjusted for patient age, gender, race, APR-DRG Severity of Illness and Risk of Mortality, year of admission, admitting physician type, hospital type (academic), hospital location (urban/rural), and number of beds. Oral Acetaminophen is the reference group.

### Total hospitalization costs

**Fig 2:**
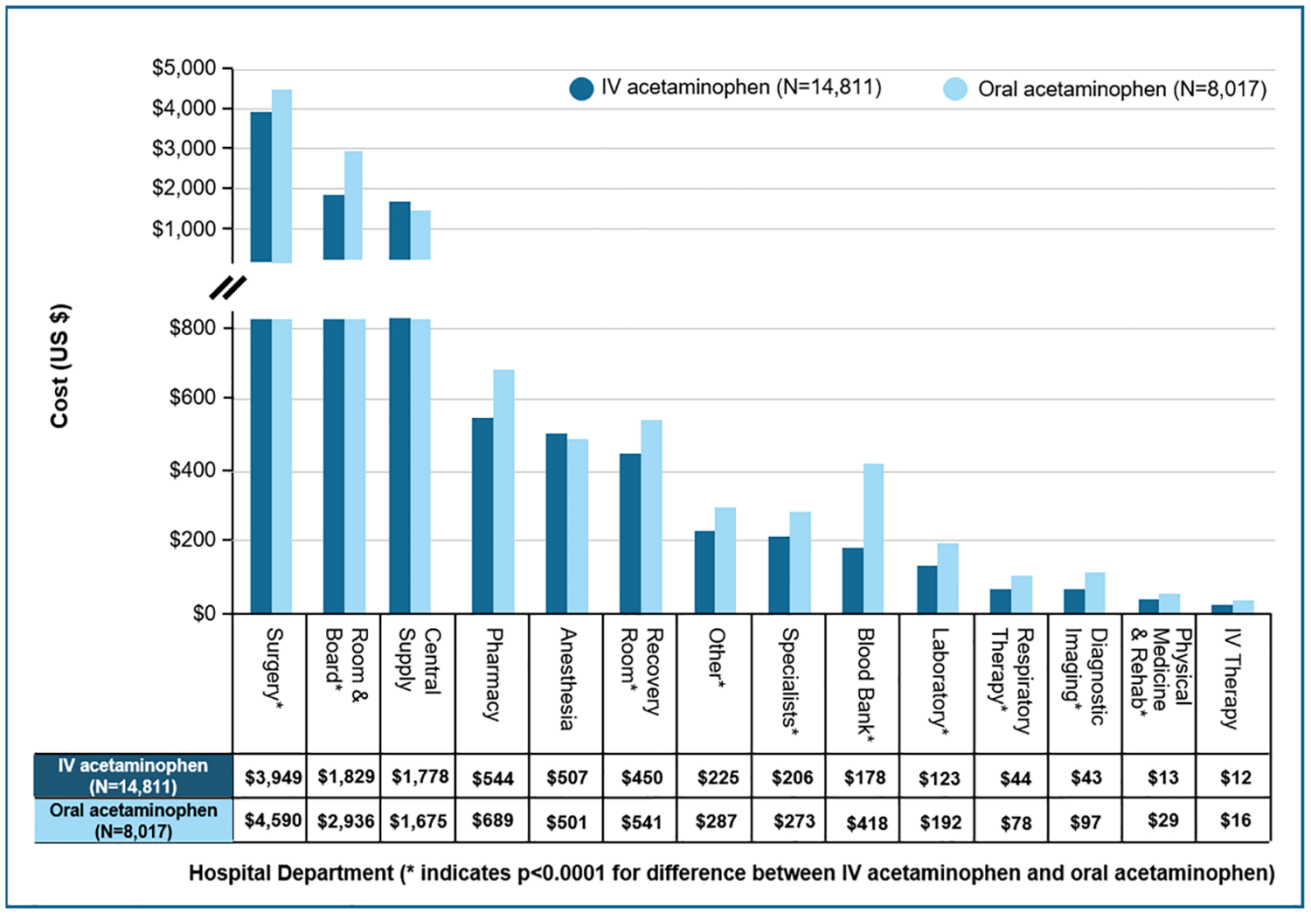
Unadjusted mean costs by Hospital Department.

We found an unadjusted total hospitalization cost difference of $2,348 lower costs for IV APAP patients compared to oral APAP patients (95% CI: -2,665 to -2,032, p<0.0001), with the costs for IV APAP patients at $9,867 (SD $7,367) compared to $12,216 (SD $13,407) for oral APAP patients (Table 2). Evaluating those average costs by hospital department revealed that IV APAP recipients had lower unadjusted costs compared to oral APAP recipients among the blood bank, laboratory, diagnostic imaging, respiratory therapy, surgery, room & board, recovery room, other specialists, physical medicine & rehabilitation, and other costs (all p<0.0001), with the largest differences observed in room & board ($1,106), surgery ($641), and blood bank ($240) (Fig 2). Our two-stage instrumental variable regression of hospitalization cost calculated a $2,449 lower cost for IV APAP recipients compared to oral APAP recipients (95% CI: -$2,902 to -$1,996, p<0.0001) (Table 3).

### Opioid consumption

Our IV APAP patients used 1.7 mg lower MED on average per day compared to oral APAP patients (95% CI: -2.8 to -0.6, p = 0.0011), with IV APAP patients using 24.9 mg MED daily on average (SD 44.7) while oral APAP patients used 26.6 mg MED daily (SD 31.7) (Table 2). Both IV and oral APAP recipients had high rates of IV fentanyl, IV hydromorphone, and IV ketorolac use as well as oral oxycodone in combination with either APAP or aspirin, ibuprofen, and hydrocodone in combination with APAP (Table 4). After applying the instrumental variable regression, the difference in opioid doses remained at 1.4 mg MED daily lower for IV APAP patients compared to oral APAP patients, but was no longer statistically significant (95% CI: -3.4 to 0.6) (Table 3).

**Table 4:** Comparison of other analgesics used from day of surgery to discharge among hysterectomy patients.

	IV Acetaminophen (n = 14,811)		Oral Acetaminophen (n = 8,017)	
**Other IV Analgesics**	n	%	n	%
Fentanyl	12182	82.25%	5277	65.82%
Hydromorphone	10491	70.83%	5073	63.28%
Ketorolac	8944	60.39%	3888	48.50%
Morphine	6393	43.16%	3103	38.71%
Meperidine	2149	14.51%	852	10.63%
**Other Oral Analgesics**	n	%	n	%
Oxycodone+Acetaminophen or Aspirin	5790	39.09%	3153	39.33%
Ibuprofen	5156	34.81%	2918	36.40%
Hydrocodone + Acetaminophen	4157	28.07%	1317	16.43%
Oxycodone	930	6.28%	1722	21.48%
Hydromorphone	691	4.67%	693	8.64%

Other IV analgesics with <5% use in either group: ibuprofen, nalbuphine, dihydroergotamine, butorphanol, methadone, buprenorphine, fentanyl/droperidol, oxymorphone, meperidine/promethazine, pentazocine. Other oral analgesics with <5% use in either group: tramadol, ibuprofen, hydromorphone, acetaminophen/codeine, morphine, methadone, acetaminophen/caffeine/butalbital, ketorolac, hydrocodone/ibuprofen, tapentadol, acetaminophen/phenyltoloxamine, tramadol/acetaminophen, codeine, acetaminophen/aspirin/caffeine, meperidine, fentanyl, acetaminophen/diphenhydramine, aspirin/caffeine/butalbital, buprenorphine, isometheptine/dich/acetaminophen, acetaminophen/caffeine/butalbital/codeine, diflunisal, oxymorphone, salsalate, levorphanol, pentazocine/acetaminophen, pentazocine/naloxone, propoxyphene/acetaminophen. Each subject was allowed to contribute up to once per other analgesic. The percentages presented use the whole study group (IV or oral acetaminophen) as the denominator.

### Complications

Our IV APAP hysterectomy population had significantly lower rates of bowel obstruction (O.R. 0.45, 95% CI: 0.38 to 0.52), nausea and vomiting (O.R. 0.62, 95% CI: 0.52 to 0.74), and respiratory depression (O.R. 0.47, 95% CI: 0.41 to 0.54) compared to oral APAP patients (all p<0.0001). The incidence of respiratory depression was also significantly different when we categorized the complication by diagnosis code, use of mechanical ventilation, and administration of naloxone (p<0.0001, 0.0002, and <0.0001, respectively–Table 2). The rates of these complications were also significantly lower for IV APAP recipients compared to oral APAP recipients in multivariable adjusted analyses (Table 5).

**Table 5:** Multivariable logistic regression comparing IV and oral acetaminophen patients*.

	Odds Ratio	95% Confidence Interval		p-value
*Complications*				
Bowel obstruction	0.69	0.58	0.81	<0.0001
Nausea/vomiting	0.67	0.55	0.81	<0.0001
Respiratory depression	0.71	0.60	0.83	<0.0001

*Adjusted for patient age, gender, race, APR-DRG Severity of Illness and Risk of Mortality, year of admission, admitting physician type, hospital type (academic), hospital location (urban/rural), and number of beds. Oral acetaminophen is the reference group.

### Hysterectomy subgroups

Upon stratification by the type of hysterectomy, using instrumental variable regression we observed that the differences in total hospitalization costs and LOS were highest among laparoscopic patients at -$3,416 (95% CI: -3,963 to -2,869, p<0.0001) and -0.74 days (95% CI: -0.87 to -0.60, p<0.0001) for IV APAP recipients compared to oral APAP recipients. The cost and LOS differences among total abdominal and vaginal hysterectomy patients were attenuated and while still statistically significant in vaginal hysterectomy patients, the differences were not significant for total abdominal hysterectomy patients (Table 6). The differences in opioid dose were also mixed, with laparoscopic and total vaginal hysterectomy patients having consistently lower daily doses in the IV APAP cohort at -2.5 mg (95% CI: -5.0 to -0.1, p = 0.043) and -3.1 mg (95% CI: -5.7 to -0.5, p = 0.019) MED, respectively. The difference in daily MED was not statistically significant among total abdominal hysterectomy patients (4.0 mg MED, 95% CI: -2.2 to 10.2, p = 0.2) (Table 6).

**Table 6:** Instrumental variable regressions comparing IV and oral acetaminophen patients, stratified by surgical approach (laparoscopic, total abdominal, or vaginal hysterectomy*.

	Laparoscopic Hysterectomy (N = 12,859)				Total Abdominal Hysterectomy (N = 4,946)				Vaginal Hysterectomy (N = 5,023)			
	Difference	95% Confidence Interval		p-value	Difference	95% Confidence Interval		p-value	Difference	95% Confidence Interval		p-value
Length of Stay (days)	-0.74	-0.87	-0.60	<0.0001	-0.32	-0.72	0.07	0.1	-0.47	-0.62	-0.32	<0.0001
Hospitalization Cost ($)	-3416.2	-3963.0	-2869.4	<0.0001	-823.8	-1821.6	174.0	0.1	-2352.1	-3507.5	-1196.7	<0.0001
Daily Morphine Equivalent Dose (mg)	-2.53	-4.97	-0.08	0.043	4.00	-2.15	10.15	0.2	-3.11	-5.70	-0.52	0.019

*Two-stage least squares with quarterly rate of IV acetaminophen use at the hospital as the instrument. Adjusted for patient age, gender, race, APR-DRG Severity of Illness and Risk of Mortality, year of admission, admitting physician type, hospital type (academic), hospital location (urban/rural), and number of beds. Oral acetaminophen is the reference group.

## Discussion

In our pooled analysis of the various surgical subgroups, managing post-hysterectomy pain with regimens including IV APAP was associated with shorter LOS and lower hospitalization costs compared to regimens that instead used oral APAP. Average daily MED trended lower in the IV APAP cohort without statistical significance. The reduction in LOS of nearly one day and associated cost savings of almost $2,500 are meaningful differences for hospitals in terms of efficiency and the opportunity to serve more patients. Additionally, pain management including IV APAP was associated with lower rates of respiratory depression, bowel obstruction, and nausea/vomiting, which represent improved outcomes that benefit both patients and the hospitals caring for them. These outcomes are of great importance in the setting of enhanced recovery programs, by affording clinicians a strategy to reduce length of stay. And our findings support advancement toward the Triple Aim by improving the patient experience and reducing health care costs [3].

In our subgroup analysis, the reduction in LOS was more pronounced in the laparoscopic surgery group, which was also associated with the greatest cost savings. Women undergoing vaginal and laparoscopic hysterectomy also had statistically significantly reduced mean daily MED associated with the use of IV APAP, but the differences between groups were small. These results contrast with a non-significant difference in 24-hour opioid consumption reported by a recent randomized clinical trial comparing IV versus oral APAP in laparoscopic cholecystectomy [30]. As acknowledged by the authors, the power to detect significant treatment differences was low due to the small sample size. Nevertheless, although a 3 mg dose-response relationship has been proposed as clinically meaningful after reaching a certain threshold [31], the relationship between MED and opioid-related adverse events warrants further investigation. Our adjusted models resulted in significantly lower rates of complications even when non-statistically significant average daily MED was observed.

We have found analogous results in our studies of IV versus oral APAP in patients undergoing spine surgery, cholecystectomy, and total knee arthroplasty [32–34]. All three of those studies found shorter length of stay, lower total hospitalization costs, and smaller opioid doses associated with IV versus oral APAP. These findings add to the real world evidence estimating the comparative effectiveness of IV versus oral APAP. Previous research of multimodal pain management including IV APAP versus opioid monotherapy also suggests that IV APAP for acute postoperative pain improves patient outcomes and reduces hospital resource use [35–39]. As health care practitioners move toward multimodal approaches to synergize distinct pain medications, increase analgesia, and reduce adverse events [2], this real world evidence is critical to help inform providers and hospital administrators about the clinical and economic benefits of IV APAP.

### Limitations

This study is subject to a number of limitations. First, the populations compared in each cohort were not randomly assigned and, although we applied techniques in an attempt to control for selection bias, other unmeasured confounding of the association may still be present. For example, one such source of confounding could be other analgesic medications that were used by the two cohorts. It is possible that differential use of another analgesic between the two groups could exacerbate or reduce the signals we observed. However, we observed that the two groups were relatively similar in the proportions of patients receiving various forms of IV and oral pain medication.

Second, the Premier Database has a few unique limitations shared with all observational or administrative claim databases. The information related to administered medications is based on charges. As such, the actual dose of medications administered was not available. While it is accepted that utilization of opioids is likely lower than recorded based on Premier hospital audits, we do not expect any systematic differences between patients who receive IV versus oral APAP and thus the difference between our two groups of interest is expected to be a valid estimate. Third, although the database consists of an approximately 20% sample of inpatient discharges in the U.S., participating hospitals are not randomly sampled and some populations are likely under-represented. However, based on the large sample sizes in each cohort, we believe that this study represents the largest possible inpatient sample that spans both the private and publicly insured (Medicaid and Medicare) populations.

### Conclusion

Compared to oral APAP, managing post-hysterectomy pain with regimens including IV APAP is associated with shorter LOS, decreased total hospitalization costs, and reduced risk of complications. Incorporation of IV APAP in a multimodal postoperative pain management regimen is recommended.
